# Ultrasound‐Guided Erector Spinae Plane Block Versus Transversus Abdominis Plane Block for Postoperative Analgesia in Adult Patients Undergoing Laparoscopic Appendectomy

**DOI:** 10.1155/anrp/6654154

**Published:** 2026-05-22

**Authors:** Eman Abdelnaby Mohamed Soliman, Mohsen Abdelgani Basyoni, Amira Fathy Hafni Soliman, Mohamed Abdelmawla Saleh, Engy Sami Attia Sami

**Affiliations:** ^1^ Assistant Lecturer of Anaesthesia, Intensive Care and Pain Management Faculty of Medicine, Ain Shams University, Cairo, Egypt, asu.edu.eg; ^2^ All from the Department of Anaesthesia, Intensive Care and Pain Management, Faculty of Medicine, Ain Shams University, Cairo, Egypt, asu.edu.eg

**Keywords:** erector spinae plane block, laparoscopic appendectomy, postoperative analgesia, regional anesthesia, transversus abdominis plane block

## Abstract

**Background:**

Effective postoperative analgesia is essential after laparoscopic appendectomy. This randomized, double‐blind controlled study compared the analgesic efficacy of ultrasound‐guided erector spinae plane block (ESP) and the transversus abdominis plane block (TAP).

**Methods:**

This randomized, double‐blind controlled study included 72 adult patients undergoing laparoscopic appendectomy. The primary outcome was the 24 h postoperative Numerical Rating Scale (NRS) pain score. Secondary outcomes included time to first rescue analgesia, total 24 h pethidine consumption, intraoperative fentanyl requirements, and adverse events. Patients were randomized to receive either an ultrasound‐guided erector spinae plane block or a transversus abdominis plane block.

**Results:**

The erector spinae plane block group demonstrated significantly lower postoperative pain scores (mean difference −0.8 to −1.4), longer time to first rescue analgesia (mean difference 1.6 hours; 95% CI: 1.2−2.0), and reduced 24‐hour pethidine consumption (mean difference −43.1 mg; 95% CI: −54.0 to −32.1). No major complications occurred.

**Conclusion:**

The erector spinae plane block provides superior postoperative analgesia after laparoscopic appendectomy and supports its inclusion in multimodal analgesic pathways.

**Trial Registration:** ClinicalTrials.gov: NCT06220513

## 1. Introduction

Although laparoscopic appendectomy is considered a minimally invasive procedure, patients may still experience considerable postoperative pain. This pain is mainly attributed to visceral irritation, surgical manipulation of the peritoneum, trauma at trocar insertion sites, and diaphragmatic stretching caused by pneumoperitoneum [1, 2].

Effective postoperative analgesia is essential not only for patient comfort but also for promoting early ambulation, reducing postoperative complications, and minimizing opioid‐related adverse effects. In recent years, ultrasound‐guided regional anesthesia techniques have gained increasing popularity due to their efficacy and safety profiles.

The transversus abdominis plane (TAP) block is a commonly used regional anesthetic technique that provides somatic analgesia to the anterior abdominal wall by blocking the thoracolumbar nerves within the fascial plane between the internal oblique and transversus abdominis muscles. However, because it mainly targets somatic nerves, its ability to control visceral pain is relatively limited [3].

The erector spinae plane (ESP) block is a relatively recent fascial plane block first described by Forero and colleagues in 2016. Injection of local anesthetic deep to the erector spinae muscle allows craniocaudal spread along the fascial plane and may reach the dorsal and ventral rami of the spinal nerves as well as the rami communicants. This distribution is believed to provide both somatic and visceral analgesic effect.

To the best of our knowledge, this randomized controlled trial provides focused, procedure‐specific evidence by comparing ultrasound‐guided ESP block and TAP block in a homogeneous population undergoing laparoscopic appendectomy with standardized perioperative management.

This study aims to compare the analgesic efficacy of ultrasound‐guided ESP block versus TAP block for postoperative pain management in adult patients undergoing laparoscopic appendectomy.

## 2. Patients and Methods

### 2.1. Sample Size Calculation

An a priori sample size calculation was performed before patient enrollment using Power Analysis and Sample Size software (PASS 15, version 15.0.10). The calculation was based on the primary outcome, namely, the 24‐h postoperative Numerical Rating Scale (NRS) pain score.

Based on the results reported by Hassanin et al. [4], a mean difference of approximately 1 point on the NRS between ESP block and TAP block was assumed, with an estimated standard deviation of 1.3. Using a two‐sided alpha level of 0.05 and a power of 80%, a minimum of 32 patients per group was required.

To account for a potential dropout rate of 10%, the final target sample size was increased to 36 patients per group, resulting in a total sample size of 72 patients.

### 2.2. Randomization and Allocation Concealment

Patients were randomly assigned to one of the two study groups in a 1:1 allocation ratio using a computer‐generated random sequence. Randomization was performed using block randomization with variable block sizes to ensure balanced group allocation throughout the study period. No stratification variables were applied.

The randomization sequence was generated by an independent anesthesiologist who was not involved in patient recruitment, block performance, intraoperative management, or postoperative outcome assessment.

Allocation concealment was achieved using sealed, opaque, sequentially numbered envelopes, which were opened only after patient enrollment and immediately before block performance.

Patient enrollment was carried out by a member of the research team, while group assignment was revealed only to the anesthesiologist responsible for performing the regional block.

### 2.3. Blinding

This study was designed as a double‐blind randomized controlled trial. Patients were blinded to group allocation and were not informed whether they received an ESP block or a TAP block.

Due to the nature of the intervention, the anesthesiologist performing the regional block was necessarily aware of group allocation but was not involved in intraoperative anesthetic management, postoperative pain assessment, data collection, or statistical analysis.

Postoperative outcome assessors, including those recording NRS pain scores and opioid consumption, were blinded to group allocation.

Data analysis was performed by a statistician who was blinded to group assignment, with groups coded prior to analysis.

### 2.4. General Anesthesia Protocol

General anesthesia was standardized for all patients in both groups. Induction of anesthesia was achieved using propofol (2–2.5 mg/kg), fentanyl (1–2 μg/kg), and rocuronium (0.6 mg/kg) to facilitate endotracheal intubation.

Anesthesia was maintained with sevoflurane in a mixture of oxygen and air, adjusted to maintain an adequate depth of anesthesia. Additional intraoperative fentanyl boluses (0.5 μg/kg) were administered if clinically indicated based on hemodynamic responses, according to a predefined protocol applied equally to both groups. Standard monitoring was used throughout the procedure, including electrocardiography, noninvasive blood pressure, pulse oximetry, and capnography.

### 2.5. Block Techniques

#### 2.5.1. ESP Block Technique

The ESP block was performed after the induction of general anesthesia with the patient placed in the lateral decubitus position. A high‐frequency linear ultrasound probe (6–13 MHz) was positioned in a longitudinal parasagittal orientation at the T9 vertebral level to identify the transverse process, erector spinae muscle, and underlying bony landmarks.

After skin antisepsis, a 22‐gauge, 80‐mm echogenic needle was advanced using an in‐plane cranial‐to‐caudal approach until the needle tip contacted the transverse process deep to the erector spinae muscle. Following negative aspiration, 20 mL of 0.25% bupivacaine was injected, with real‐time ultrasound confirmation of linear spread in the fascial plane between the erector spinae muscle and the transverse process. The block was performed bilaterally in all patients.

#### 2.5.2. TAP Block Technique

The TAP block was performed after the induction of general anesthesia with the patient at the supine position. A high‐frequency linear ultrasound probe (6–13 MHz) was placed transversely at the midaxillary line between the costal margin and iliac crest, corresponding to the lateral TAP approach.

The external oblique, internal oblique, and transversus abdominis muscles were identified. A 22‐gauge, 80‐mm echogenic needle was advanced using an in‐plane medial‐to‐lateral approach until the needle tip was positioned in the fascial plane between the internal oblique and transversus abdominis muscles. After negative aspiration, 20 mL of 0.25% bupivacaine was injected on each side, with ultrasound confirmation of appropriate hydrodissection of the target plane.

### 2.6. Assessment of Block Success

Formal sensory testing was not feasible due to general anesthesia; therefore, block success was assessed indirectly based on intraoperative hemodynamic stability, reduced intraoperative opioid requirements, and postoperative pain scores and analgesic consumption. No technical difficulties were encountered during block performance, and no block failures or need for additional regional techniques were recorded.

### 2.7. Postoperative Pain Assessment

Postoperative pain was assessed using the NRS, where 0 indicates no pain and 10 indicates the worst imaginable pain. Before surgery, all patients were instructed on the use of the NRS.

Pain scores were recorded at rest and on movement at predefined postoperative time points (0.5, 2, 4, 6, 8, 12, 18, and 24 h). Pain “on movement” was defined as pain experienced during active trunk movement, such as sitting up in bed or coughing.

All NRS assessments were performed by trained postoperative nursing staff that was blinded to group allocation.

### 2.8. Postoperative Analgesia Protocol

All patients received a standardized multimodal postoperative analgesia regimen. This included intravenous paracetamol 1 g every 8 h and ibuprofen 400 mg every 8 h, unless contraindicated.

Pethidine was used as rescue analgesia and was administered as a fixed intravenous dose of 25 mg when the NRS pain score was ≥ 4 at rest. The time to the first rescue analgesic request and the total cumulative pethidine consumption during the first 24 h were recorded as secondary outcomes. No additional opioid or nonopioid rescue analgesics were permitted during the study period. Antiemetic prophylaxis was administered according to institutional protocol and was identical in both groups.

### 2.9. Definition of Adverse Events

Adverse events were predefined and included block‐related complications (local anesthetic systemic toxicity, hematoma, pneumothorax, and infection), persistent motor weakness, postoperative nausea and vomiting, hypotension, and bradycardia. Serious adverse events were defined as those requiring intervention or prolonging hospital stay.

### 2.10. Study Outcomes

The primary outcome of this randomized controlled trial was the 24‐h postoperative NRS pain score, assessed at predefined postoperative time points both at rest and during movement.

Secondary outcomes included time to first rescue analgesia, total 24‐h pethidine consumption, intraoperative fentanyl requirements, postoperative hemodynamic variables, and the incidence of block‐related or postoperative adverse events.

### 2.11. Statistical Analysis

Data were analyzed using SPSS software (Version 26). Normality of continuous variables was assessed using the Shapiro–Wilk test. Normally distributed data were analyzed using parametric tests; while nonnormally distributed data were analyzed using nonparametric tests, as appropriate. Continuous variables are presented as mean ± standard deviation and categorical variables as numbers and percentages. For the primary outcome (postoperative NRS pain scores), repeated measurements over time were analyzed using a repeated‐measures analysis of variance (ANOVA) model, with time as the within‐subject factor and group as the between‐subject factor. Post hoc comparisons were adjusted for multiple testing.

Time to first rescue pethidine dose was analyzed using Kaplan–Meier survival analysis, and groups were compared using the log‐rank test.

Secondary continuous outcomes were compared using the independent *t*‐test or Mann–Whitney *U* test, as appropriate. Categorical variables were compared using the chi‐square test or Fisher’s exact test. Effect sizes were expressed as mean differences with 95% confidence intervals. A two‐sided *p* value < 0.05 was considered statistically significant.

### 2.12. Ethical Approval and Registration

**FIGURE 1:**
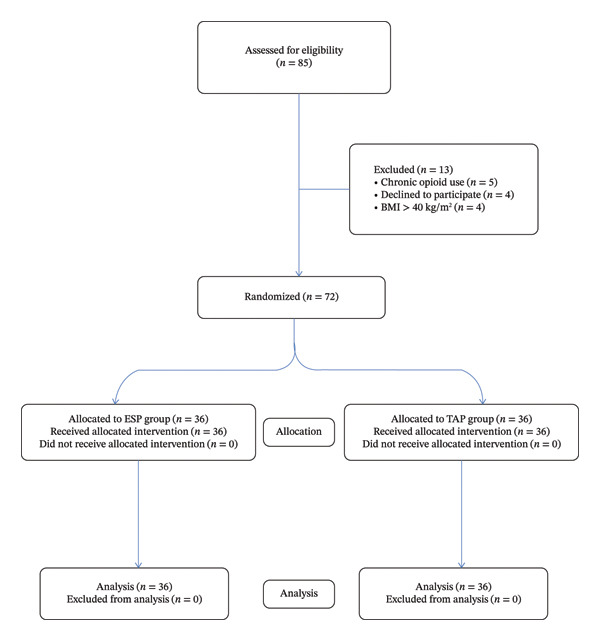
CONSORT flow diagram of patient enrollment, randomization, allocation, follow‐up, and analysis.

Ethical approval for this study was obtained from the Research Ethics Committee of the Faculty of Medicine, Ain Shams University, Cairo, Egypt (approval number: FMASU MD229/2023, approval date: 9 September 2023). The study was conducted in accordance with the principles of the Declaration of Helsinki and followed the guidelines of the International Council on Harmonization and the Islamic Organization for Medical Sciences. Written informed consent was obtained from all participants prior to enrollment. Following approval by the Research Ethics Committee in September 2023, patient recruitment was conducted from January 2024 to July 2025. (see Figure 1).

## 3. Results

No statistical significant difference was found between the studied groups regarding age, gender, BMI, and ASA class (Table 1).

**TABLE 1:** Demographic characteristics between the study groups.

Variables		ESP group (total = 36)	TAP group (total = 36)	*p* value
Age (years)		27.1 ± 3.9	28.5 ± 4.5	0.185

Gender (*n*, %)	Male	22 (61.1%)	25 (69.4%)	0.458
	Female	14 (38.9%)	11 (30.6%)	

BMI (kg/m^2^)		28.6 ± 3.8	29.1 ± 2.8	0.544

ASA (*n*, %)	I	19 (52.8%)	21 (58.3%)	0.635
	II	17 (47.2%)	15 (41.7%)	

No statistical significant difference was found between the studied groups regarding operation duration, anesthesia duration, and hematoma. Local anesthetic toxicity, intravenous injection, nerve injury, and need to intraoperative fentanyl did not occur in either group (Table 2).

**TABLE 2:** Operative events between the study groups.

Variables	ESP group (total = 36)	TAP group (total = 36)	*p* value	Effect size	
				Mean ± SE/relative risk	95% CI
Operation duration (min)	82.8 ± 18.6	86.9 ± 14.8	0.297	−4.2 ± 4.0	−12.1–3.7
Anesthesia duration (min)	96.5 ± 19.0	100.8 ± 14.9	0.289	−4.3 ± 4.0	−12.3–3.7
Hematoma	6 (16.7%)	5 (13.9%)	0.743	1.20	0.40–3.58
Local anesthetic toxicity	0 (0.0%)	0 (0.0%)	NA	NA	NA
Intravenous injection	0 (0.0%)	0 (0.0%)	NA	NA	NA
Nerve injury	0 (0.0%)	0 (0.0%)	NA	NA	NA

*Note:* Data are presented as mean ± SD or number (%). Effect size: effect in the ESP group relative to that in the TAP group.

Abbreviations: CI, confidence interval; NA, not applicable; SE, standard error.

No statistical significant difference was found between the study groups regarding intraoperative heart rate and mean arterial pressure (Table 3).

**TABLE 3:** Intraoperative heart rate and mean arterial pressure between the study groups.

Intraoperative time	ESP group (total = 36)	TAP group (total = 36)	*p* value	Effect size	
				Mean ± SE	95% CI
Heart rate (beat/min)					
Baseline	83.5 ± 2.6	82.9 ± 1.9	0.261	0.6 ± 0.5	−0.5–1.7
Induction	87.4 ± 2.7^#^	86.6 ± 1.9^#^	0.126	0.9 ± 0.6	−0.2–2.0
Min‐10	93.3 ± 2.7^#^	92.4 ± 2.2^#^	0.124	0.9 ± 0.6	−0.3–2.1
Min‐20	77.8 ± 2.6^#^	77.3 ± 2.3^#^	0.392	0.5 ± 0.6	−0.7–1.7
Min‐30	75.2 ± 2.5^#^	74.9 ± 1.8^#^	0.552	0.3 ± 0.5	−0.7–1.3
Min‐40	74.3 ± 2.3^#^	73.5 ± 2.1^#^	0.129	0.8 ± 0.5	−0.2–1.9
Min‐50	71.9 ± 2.4^#^	71.5 ± 2.1^#^	0.411	0.4 ± 0.5	−0.6–1.5
Min‐60	71.8 ± 2.5^#^	71.2 ± 2.0^#^	0.329	0.5 ± 0.5	−0.5–1.6
Operation end	70.2 ± 2.2^#^	69.5 ± 1.8^#^	0.147	0.7 ± 0.5	−0.3–1.6
Mean arterial pressure (mmHg)					
Baseline	93.8 ± 2.9	93.2 ± 2.2	0.319	0.6 ± 0.6	−0.6–1.8
Induction	98.2 ± 3.2	97.0 ± 2.4	0.074	1.2 ± 0.7	−0.1–2.6
Min‐10	104.5 ± 3.2	103.5 ± 2.5	0.163	0.9 ± 0.7	−0.4–2.3
Min‐20	87.2 ± 3.0	86.8 ± 2.6	0.615	0.3 ± 0.7	−1.0–1.7
Min‐30	84.3 ± 2.8	84.1 ± 2.1	0.812	0.1 ± 0.6	−1.0–1.3
Min‐40	83.3 ± 2.6	82.5 ± 2.4	0.200	0.8 ± 0.6	−0.4–2.0
Min‐50	81.0 ± 3.1	80.4 ± 2.4	0.427	0.5 ± 0.7	−0.8–1.8
Min‐60	80.7 ± 2.8	79.9 ± 2.4	0.223	0.8 ± 0.6	−0.5–2.0
Operation end	79.0 ± 2.5	78.1 ± 2.1	0.108	0.9 ± 0.5	−0.2–2.0

*Note:* Data are presented as mean ± SD. Effect size: effect in the ESP group relative to that in the TAP group.

Abbreviations: CI, confidence interval; SE, standard error.

^#^Significantly different from baseline.

**FIGURE 2:**
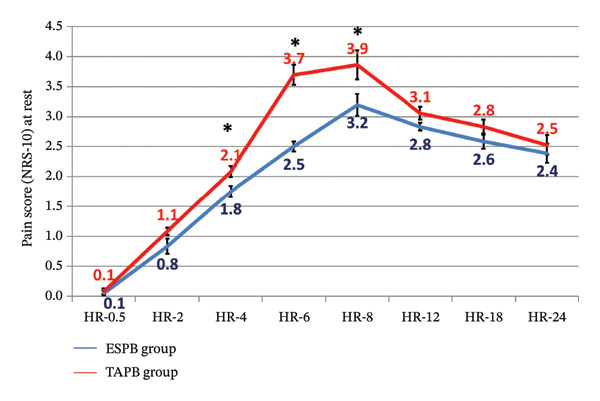
Postoperative pain score (NRS‐10) at rest between the study groups (^∗^significant).

**FIGURE 3:**
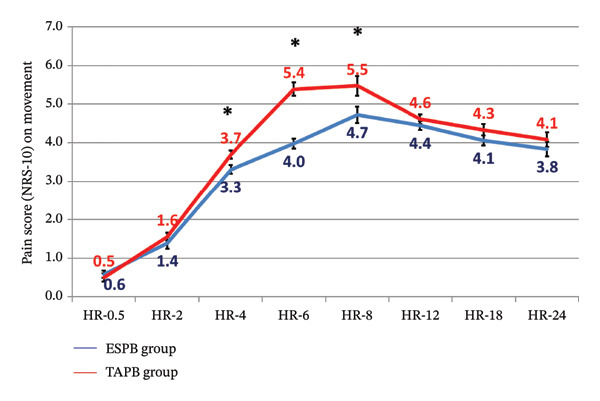
Postoperative pain score (NRS‐10) on movement between the study groups (^∗^significant).

Pain scores at rest and on movement were significantly lower among the ESP group from hour 4 until hour 8 postoperatively (Table 4 and Figures 2 and 3).

**TABLE 4:** Postoperative pain score (NRS‐10) between the study groups.

Postoperative time	ESP group (total = 36)	TAP group (total = 36)	*p* value	Effect size	
				Mean ± SE	95% CI
At rest					
Hr‐0.5	0.1 ± 0.2	0.1 ± 0.3	0.649	0.0 ± 0.1	−0.1–0.1
Hr‐2	0.8 ± 0.7	1.1 ± 0.4	0.073	−0.3 ± 0.1	−0.5–0.0
Hr‐4	1.8 ± 0.6	2.1 ± 0.6	**0.013** ^**∗**^	−0.3 ± 0.1	−0.6–−0.1
Hr‐6	2.5 ± 0.5	3.7 ± 1.0	**0.001** ^**∗**^	−1.2 ± 0.2	−1.6–−0.8
Hr‐8	3.2 ± 1.1	3.9 ± 1.5	**0.031** ^**∗**^	−0.7 ± 0.3	−1.3–−0.1
Hr‐12	2.8 ± 0.4	3.1 ± 0.6	0.074	−0.2 ± 0.1	−0.5–0.0
Hr‐18	2.6 ± 0.7	2.8 ± 0.7	0.131	−0.3 ± 0.2	−0.6–0.1
Hr‐24	2.4 ± 1.0	2.5 ± 1.0	0.544	−0.1 ± 0.2	−0.6–0.3
On movement					
Hr‐0.5	0.6 ± 0.6	0.5 ± 0.6	0.546	0.1 ± 0.1	−0.2–0.4
Hr‐2	1.4 ± 0.9	1.6 ± 0.6	0.361	−0.2 ± 0.2	−0.5–0.2
Hr‐4	3.3 ± 0.7	3.7 ± 0.6	**0.013** ^**∗**^	−0.4 ± 0.2	−0.7–−0.1
Hr‐6	4.0 ± 0.8	5.4 ± 1.0	**< 0.001** ^**∗**^	−1.4 ± 0.2	−1.9–−1.0
Hr‐8	4.7 ± 1.3	5.5 ± 1.5	**0.027** ^**∗**^	−0.8 ± 0.3	−1.4–−0.1
Hr‐12	4.4 ± 0.7	4.6 ± 0.7	0.310	−0.2 ± 0.2	−0.5–0.2
Hr‐18	4.1 ± 0.8	4.3 ± 0.9	0.167	−0.3 ± 0.2	−0.7–0.1
Hr‐24	3.8 ± 1.1	4.1 ± 1.1	0.341	−0.3 ± 0.3	−0.8–0.3

*Note:* Data are presented as mean ± SD. Effect size: effect in the ESPB group relative to that in the TAPB group. Bold values indicate statistical significance.

Abbreviations: CI, confidence interval; SE, standard error.

^∗^Significant.

**FIGURE 4:**
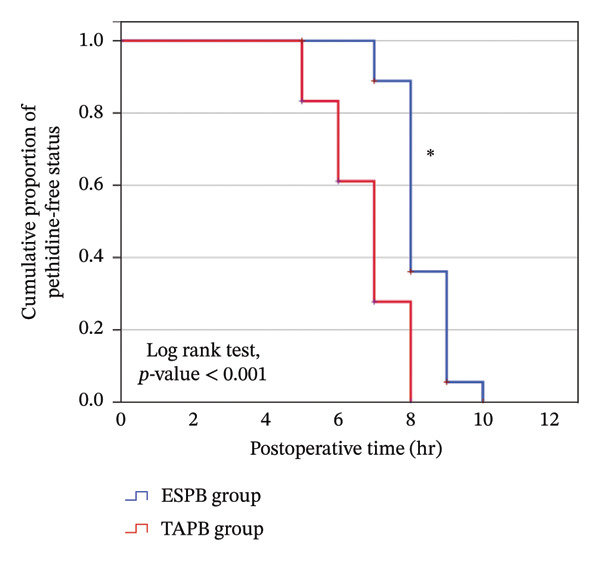
Kaplan–Meier curve for the rate of first need to pethidine between the study groups (^∗^significant).

The ESP group had significant longer time to first pethidine dose and lower total 24‐h pethidine dose (Table 5). Rate of requesting first pethidine dose was significantly lower in the ESP group (Figure 4).

**TABLE 5:** Postoperative pethidine requirements between the study groups.

Variables	ESP group (total = 36)	TAP group (total = 36)	*p* value	Effect size	
				Mean ± SE	95% CI
Time to first pethidine dose (hr)	8.3 ± 0.7	6.7 ± 1.1	< 0.001**^∗^**	1.6 ± 0.2	1.2–2.0
Total 24‐h pethidine dose (mg)	119.4 ± 24.7	162.5 ± 22.0	< 0.001**^∗^**	−43.1 ± 5.5	−54.0–‐32.1

*Note:* Data are presented as mean ± SD or number (%). Effect size: effect in the ESPB group relative to that in the TAPB group. Bold values indicate statistical significance.

Abbreviations: CI, confidence interval; SE, standard error.

^∗^Significant.

No significant difference was found between the studied groups regarding intraoperative fentanyl requirements (Table 6).

**TABLE 6:** Intraoperative fentanyl requirements between the study groups.

Variables	ESP group (total = 36)	TAP group (total = 36)	*p* value	Effect size	
				Mean ± SE/relative risk	95% CI
Intraoperative fentanyl	7 (19.4%)	8 (22.2%)	0.772	0.88	0.35–2.16
Total fentanyl dose (μg)	92.9 ± 34.5	118.8 ± 37.2	0.188	−25.9 ± 18.6	−66.1–14.3

*Note:* Data are presented as mean ± SD. Effect size: effect in the ESP group relative to that in the TAP group.

Abbreviations: CI, confidence interval; SE, standard error.

### 3.1. Postoperative Adverse Events

Postoperative adverse events were assessed during the first 24 h after surgery. The incidence of postoperative nausea and vomiting was recorded in both groups, with no statistically significant difference between them. No cases of pruritus, excessive sedation, respiratory depression, urinary retention, or block‐related complications were observed in either group.

## 4. Discussion

Laparoscopic appendectomy, although minimally invasive, can still result in significant postoperative pain due to visceral traction, peritoneal irritation, and pneumoperitoneum‐induced diaphragmatic stretching. Effective postoperative analgesia is, therefore, essential to optimize patient recovery, reduce opioid exposure, and facilitate early mobilization.

In this randomized controlled trial, the blocks were performed preoperatively to achieve pre‐emptive analgesia, aiming to attenuate central sensitization and reduce early postoperative pain and opioid consumption.”

The ESP block demonstrated both clinically meaningful and statistically significant superiority over the TAP block, with consistent benefits across all major pain‐related outcomes and differences exceeding the minimum clinically important difference reported for postoperative pain scales. Patients in the ESP group exhibited lower pain scores, delayed need for rescue analgesia, and reduced total 24‐h pethidine consumption. The magnitude of these differences aligns with clinically relevant analgesic benefit, rather than statistical significance alone.

The mechanistic basis for these findings is supported by the original description of the ESP block by Forero et al. [5], who demonstrated that local anesthetic deposited deep to the erector spinae muscle can spread cranially and caudally along the fascial plane.

The analgesic mechanism of the ESP block is thought to involve diffusion of local anesthetic along the fascial plane deep to the erector spinae muscle, with potential spread to the paravertebral region. This spread may influence both somatic and sympathetic nerve pathways, which could explain the improved visceral pain control observed with this block [6]. This broad pattern of distribution allows the ESP block to provide both somatic and visceral analgesia. In contrast, the TAP block predominantly offers somatic analgesia of the anterior abdominal wall, which may be insufficient for procedures involving peritoneal stretching.

Our results are also consistent with emerging evidence from high‐quality meta‐analyses and randomized trials. Oraee et al. [7] demonstrated that the ESP block significantly reduces postoperative opioid consumption and pain scores across several laparoscopic abdominal procedures. Similarly, Qian et al. [8] confirmed that the ESP block provides more effective visceral analgesia and broader dermatomal coverage compared with the TAP block. In addition, the randomized controlled trial by Park et al. [9] in laparoscopic colorectal surgery showed reduced opioid requirements and faster mobilization when ESP block was incorporated into enhanced recovery pathways. These collective findings strongly support the expanding role of ESP block in abdominal surgery.

Unlike previously published systematic reviews and meta‐analyses comparing ESP block and TAP block across a wide range of abdominal surgical procedures, the present study specifically focuses on a homogeneous surgical population undergoing laparoscopic appendectomy. The meta‐analysis published in the Journal of Investigative Surgery (Vol 35, No 9) [10] included diverse abdominal surgeries with variable incision sites, anesthetic techniques, and postoperative analgesic regimens, which may contribute to clinical heterogeneity and limit procedure‐specific conclusions. In contrast, our randomized controlled trial minimized such variability by applying standardized anesthesia and multimodal analgesia protocols, thereby allowing a more precise assessment of block‐related analgesic efficacy. Furthermore, our study reports effect sizes and time‐dependent pain assessments, demonstrating clinically relevant differences rather than statistical significance alone. These findings provide procedure‐specific evidence supporting the superiority of the ESP block for postoperative analgesia following laparoscopic appendectomy. Regarding safety, no major block‐related complications were observed in this study. This aligns with current ESP literature, which reports a low incidence of significant adverse events and supports the block’s favorable safety profile.

### 4.1. Strengths and Limitations

This study has several strengths, including its prospective randomized design, standardized general anesthesia and postoperative analgesia protocols, and the evaluation of clinically relevant outcomes such as pain scores and opioid consumption.

However, some limitations should be acknowledged. This was a single‐center study with a relatively small sample size, which may limit the generalizability of the results. Blinding of the anesthesiologist performing the block was not feasible, and opioid‐related side effects were recorded clinically without the use of a structured scoring system. In addition, long‐term outcomes beyond the first 24 postoperative hours were not assessed.

## 5. Conclusion

The ultrasound‐guided ESP block is more effective than the TAP block for postoperative analgesia in adult patients undergoing laparoscopic appendectomy. The ESP block significantly reduces pain scores during the early postoperative period, delays the need for rescue analgesia, and lowers total opioid consumption, all while maintaining a favorable safety profile.

Given these findings, the ESP block may be a valuable component of multimodal analgesia protocols for laparoscopic abdominal surgeries. Further large‐scale, multicenter trials are warranted to validate these results and to explore the benefits of continuous ESP block techniques in enhancing postoperative recovery and patient satisfaction.

## Author Contributions

Eman Abdelnaby Mohamed Soliman contributed to study design, patient recruitment, data collection, data analysis, and manuscript drafting.

Prof. Mohsen Abdelgani Basyoni contributed to study supervision and critical revision of the manuscript.

Prof. Amira Fathy Hafni Soliman contributed to study design and scientific supervision.

Dr. Mohamed Abdelmawla Saleh contributed to methodological guidance and manuscript revision.

Dr. Engy Sami Attia Sami contributed to data interpretation and manuscript revision.

## Funding

This research received no external funding.

## Disclosure

All authors have reviewed and approved the final manuscript.

## Conflicts of Interest

The authors declare no conflicts of interest.

## Data Availability

The datasets generated and analyzed during the current study are available from the corresponding author upon reasonable request.
